# Effect of metoprolol exposure following myocardial infarction on future cardiovascular events: a Mendelian randomization study

**DOI:** 10.1007/s00228-025-03806-w

**Published:** 2025-02-03

**Authors:** Lina Dorthea Bruun, Geir Øystein Andersen, Marianne Kristiansen Kringen, Peder Langeland Myhre, Sigrun Halvorsen, Charlotte Holst Hansen, Espen Molden, Erik Øie

**Affiliations:** 1 Department of Pharmacology, Oslo University Hospital, Ullevål, PO BOX4956, Nydalen, Oslo, N-0424 Norway; 2 Department of Cardiology, Oslo University Hospital, Ullevål, Oslo Norway; 3 Center for Psychopharmacology, Diakonhjemmet Hospital, Oslo, Norway; 4 Department of Health Sciences, Oslo Metropolitan University, Oslo, Norway; 5 Division of Medicine, Akershus University Hospital, Lørenskog, Norway; 6 K.G. Jebsen Center of Cardiac Biomarkers, Institute for Clinical Medicine, University of Oslo, Oslo, Norway; 7 Institute for Clinical Medicine, University of Oslo, Oslo, Norway; 8 Department of Pharmacy, University of Oslo, Oslo, Norway; 9 Department of Internal Medicine, Diakonhjemmet Hospital, Oslo, Norway

**Keywords:** Metoprolol, CYP2D6, Myocardial infarction, Genotype, Mendelian randomization

## Abstract

**Purpose:**

The clinical benefit of up-titration of metoprolol to a guideline-recommended target dose after myocardial infarction (MI) is unknown. Our aim was to investigate whether variation in metoprolol exposure determined by cytochrome p450 enzyme 2D6 (CYP2D6*)* influences the occurrence of major adverse cardiovascular events (MACE) and cardiovascular death (CV death) among patients treated with metoprolol after MI.

**Method:**

This Mendelian randomization study was performed using individual-level data from 1554 patients treated with metoprolol after an acute MI. CYPD26 genotype was applied as a binary genetic instrument assigning patients into two metoprolol exposure groups: CYP2D6 normal metabolizers (NM) (low exposure) and CYP2D6 intermediate and poor metabolizers (IM + PM) (high exposure). The null hypothesis of no association between the CYP2D6 metabolizer subgroup and MACE or CV death was tested using the Cox proportional hazards model. All-cause mortality and individual components of MACE were included as secondary outcomes.

**Results:**

In total, 879 (56.6%) patients were classified as NM and 675 (43.4%) as IM + PM. During the 3-year follow-up, 56 patients (6.4%) in the NM group had an outcome of MACE, and 24 (2.7%) patients died from CV disease. Corresponding frequency in the IM + PM group was 47 (7.0%) and 22 (3.3%), respectively. There was no association between genotype and MACE [unadjusted HR 1.12 (CI 0.76, 1.65)] or CV death [unadjusted HR 1.20 (CI 0.67, 2.14)], or between the CYP2D6 group and any of the secondary outcomes.

**Conclusion:**

In patients treated with metoprolol after MI, variation in metoprolol exposure determined by CYP2D6 did not impact the occurrence of cardiovascular events.

**Supplementary Information:**

The online version contains supplementary material available at 10.1007/s00228-025-03806-w.

## Introduction

International guidelines [1] recommend that long-term treatment with a beta-blocker should be considered for all patients following myocardial infarction (MI), a recommendation based on randomized controlled trials (RCT) performed in the 1980s [2–4]. The benefit of beta-blockers after MI in patients without reduced left ventricular ejection fraction (EF) and arrhythmias has been questioned due to the improvements in invasive strategies and secondary preventive pharmacotherapy [1, 5, 6]. Whilst more recent observational studies, with their inevitable risk of bias, have reported mixed results regarding the benefit of beta-blocker treatment and the importance of achieving recommended target dose [7–14], results from the just-out open-label RCT (REDUCE-AMI) did not find any benefit of beta-blocker treatment on all-cause mortality or MI among patients with preserved left ventricular EF [15].

In Norway, 82% of patients admitted to hospital with an acute MI are discharged on treatment with a beta-blocker, with a majority (72%) prescribed the ß_1_-selective beta-blocker metoprolol [16]. The majority of patients who are started on beta-blockers are still treated after 24 months [16]. While the optimal dose of metoprolol post-MI remains to be determined, the original trials provided documentation for a daily target dose of 200 mg [2]. However, metoprolol doses reported in real-world practice are substantially lower and indicate that very few patients are up-titrated to 200 mg [12, 14, 17]. We have previously reported a mean dose of 60.6 mg daily at discharge, remaining practically unchanged (62.3 mg) after 1 year [16].

Metoprolol is extensively metabolized into inactive metabolites by the highly polymorphic cytochrome P450 enzyme 2D6 (CYP2D6) [18, 19], an enzyme with more than 100 genetic variants identified [20]. *CYP2D6* genotype heavily influences the pharmacokinetics of metoprolol, contributing to large inter-individual variation in drug exposure [17, 19, 21–24]. In a study of post-MI patients, we demonstrated a six-fold variation in metoprolol plasma trough concentrations between genotypes, accompanied by an attenuation in achieved maximum heart rate among patients encoding strongly decreased or absent CYP2D6 metabolism vs. those expressing normal CYP2D6 metabolism [17]. Although genotypes with high metoprolol exposure have been demonstrated to associate with a greater reduction in heart rate and blood pressure [21, 23, 24], the clinical significance of metoprolol dose and exposure for major adverse cardiovascular events (MACE) has not been thoroughly elucidated.

In this study, we aimed to investigate whether variation in metoprolol exposure determined by *CYP2D6* genotype influenced the occurrence of MACE and cardiovascular (CV) death among post-MI patients using Mendelian randomization to test the null hypothesis of no association between genotype and cardiovascular events.

## Method

### Study design

On the premise of random inheritance of genetic variants from parent to offspring, the method of Mendelian randomization utilizes genetic variation to study the causal effects of modifiable exposures on an outcome. Genetic variants can be considered instrumental variables (IVs) assuming they are associated with the exposure, not associated with any confounders of the exposure-outcome relationship, and only associated with the outcome through the exposure [25, 26]. In this one-sample, individual-level Mendelian randomization study, we propose CYP2D6 as a candidate instrument for assigning patients into two metoprolol exposure groups.

The study was performed in line with recent guidelines for Mendelian randomization studies [27] and reported according to the STROBE-MR recommendations [28].

### Study population

Biobanking in Acute Myocardial Infarction (BAMI) was a prospective, observation study of 2150 patients admitted to Oslo University Hospital, Ullevål (Oslo, Norway) with acute MI in the period June 2007–2017. The patients were included during weekdays and gave written informed consent. Exclusion criteria were age < 18 years, unstable condition, and being unable (sedated) or not consenting to participate. Only patients discharged with metoprolol treatment were eligible for inclusion in the present study. The study flow chart is described in Supplementary material online, Fig. S1.

Baseline information on comorbid conditions and use of beta-blockers was collected from questionnaires and hospital records at the time of inclusion. Hypertension, previous MI, heart failure (HF), diabetes mellitus (DM), previous cerebrovascular conditions, and chronic kidney disease (serum-creatinine > 150 µmol/L) were defined as present/absent. Information on metoprolol doses at discharge, as well as concomitant treatment with other pre-specified drugs, was extracted from the patient discharge records. The metoprolol dose was determined by the treating physician, who had access to heart rate, blood pressure, and clinical status, according to routine practice.

The 10th International Classification of Diseases system (ICD-10) was used for all outcomes reported. Clinical outcomes and dates of recurrent MI (ICD-10, I21), HF (I50), and stroke (I60-64) were obtained by linkage to the National Patient Registry, whereas all-cause mortality, date of death, and classification of causes of death (cardiovascular or non-cardiovascular) were obtained from the Norwegian Cause-of-Death Registry.

Time zero was set to the date of inclusion during hospitalization for the index MI, and patients were followed until the first CV event, death, or end of follow-up. Duration of follow-up was restricted a priori to 3 years.

The study was approved by the Regional Committee for Medical and Health Research Ethics (Case number: 107832).

### Blood samples

Blood samples were collected after an overnight fast and subsequently biobanked and stored at − 80^∘^C. Analyses of *CYP2D6* variant alleles were performed on whole blood.

### Genotyping and metabolizer classification

Genotyping of *CYP2D6* variant alleles was performed using Taqman-based real-time PCR assays implemented for routine pharmacogenetic analyses. The *CYP2D6* pharmacogenetic panel included the non-functional (*null*) alleles CYP2D6*3 (rs35742686), CYP2D6*4 (rs3892097), CYP2D6*5 (whole gene deletion), and CYP2D6*6 (rs5030655); the reduced-function (*red*) variants CYP2D6*9 (rs5030656), CYP2D6*10 (rs1065852), and CYP2D6*41 (rs28371725); as well as copy number analysis to identify multiplication of functional alleles giving rise to ultrarapid metabolism. The absence of the assayed variant alleles was interpreted as CYP2D6*1 (wild type).

Patients were assigned into four genotype-predicted metabolizer subgroups: (i) poor metabolizers (PM) (carriers of *null*/*null* or *red*/*null* diplotypes); (ii) intermediate metabolizers (IM) (carriers of *1/*null* or *red*/*red* diplotypes); (iii) normal metabolizers (NM) (carriers of *1/*1 or *red*/*1 alleles; or (iv) ultrarapid metabolizers (UM) (carriers of three or more functional *1 alleles). Patients with duplication of alleles other than *1 were excluded. These assignments were in line with current Clinical Pharmacogenetics Implementation Consortium (CPIC) guidelines [29] for genotype-predicted subgrouping of CYP2D6 phenotypes, except from *red*/*null* diplotypes, which in guidelines are assigned to the IM subgroup but allocated to the PM group in the present study. The reason for allocating *red*/*null* diplotypes to PM was our recent study showing that CYP2D6 metabolism of metoprolol is reduced to a similar extent in patients with *null*/*null* and *red*/*null* diplotypes [17].

For the primary statistical analyses in this study, we categorized patients into a merged group of CYP2D6 IM + PM (“high metoprolol exposure”) and a NM group (“low metoprolol exposure”). In a secondary analysis, we also investigated the effect of NM vs. PM as a distinct group. Due to the limited number of patients that were UM (*n* = 34), this group was excluded.

Although available as a routine analysis, *CYP2D6* genotyping is not commonly performed before initiating metoprolol treatment post-MI. Hence, genotype was assumed not to affect study entry or metoprolol dosing, allowing for exchangeability of the metabolizer subgroups at baseline. As recommended in the absence of precise information on the factual exposure (metoprolol dose, treatment duration, and compliance), the aim was to test for a causal association between genotypes and the outcome [25, 30, 31]. There is no gold standard as how to deal with time-to-event data in Mendelian randomization, as conditioning on previous survival could introduce bias; however, null hypothesis testing of “no effect” allows for the use of standard techniques from survival analyses [30, 32, 33].

### Outcomes

Primary endpoints were (i) CV death and (ii) a composite of MACE (CV death, recurrent MI, HF, and stroke). Secondary endpoints were (i) all-cause mortality, (ii) recurrent MI, (iii) HF, and (iv) stroke.

### Statistical methods

Categorical data are presented as numbers (%) and continuous data as mean (SD) and range. A comparison of baseline characteristics was not performed. Clinical outcomes are displayed as cumulative incidence plots with risk tables. Associations between CYP2D6 and events were assessed using a Cox proportional hazards (PH) model, presented as population-level hazard ratios (HR) with corresponding 95% confidence intervals (CI) and *p*-values. Only the first event for each individual was included, and competing events (non-cardiovascular death for primary outcomes and death for secondary outcomes) were censored. Missing data are reported, with adjustment to baseline covariates performed only in complete case individuals. Adjusted models include the following covariates: sex, age, hypertension, previous MI, HF, DM, cerebrovascular conditions, and chronic kidney disease (serum-creatinine > 150 µmol/L). The effect of age was also evaluated in separate models using age as the time scale. The PH assumption was inspected visually by log-minus-log plots and tested using Schoenfeld residuals. Some deviations were accepted as within reason. Statistical significance was set at *p*-values < 0.05.

We allowed relaxation of the core assumptions to evaluate the relative heterogeneity of effects across subgroups, and performed the following subgroup analyses to further evaluate the robustness of the overall findings: (i) comparing the CYP2D6 NM vs. PM subgroup alone (without the IM metabolizer group), (ii) excluding individuals already treated with metoprolol at baseline, (iii) excluding patients with HF at baseline (a composite of established HF and/or serum N-terminal pro-B-type natriuretic peptide (NT-proBNP) > 500 ng/mL and/or left ventricular EF < 40% at baseline), and (iv) analyzing patients with ST-elevation MI (STEMI) and non-STEMI (NSTEMI) separately. We also assessed interactions between the CYP2D6 metabolizer group with the baseline variables age, sex, diabetes, the type of MI, and HF for the primary outcomes.

Based on the exploratory nature of the study, correction due to multiple testing was not performed. All data were analyzed using Stata version 17.0.

## Results

One thousand five hundred fifty-four (72.2%) patients included in the BAMI cohort were discharged on metoprolol and with a *CYP2D6* genotype categorized as NM (*n* = 879), IM (*n* = 487), or PM (*n* = 188), providing a study cohort comprising 879 patients (56.6%) in the high metoprolol–exposure group and 675 patients (43.4%) in the low metoprolol–exposure group.

In the whole study population, approximately 80% were male, the mean age was 60.9 years (range 24–94), and 83.1% of the included patients were admitted with STEMI. The distribution of baseline characteristics was similar between the NM and IM + PM groups. At discharge, the mean dose of metoprolol (extended-release metoprolol succinate) was 52.7 (± 35.0) mg in the NM group and 49.6 (± 33.0) mg in the IM + PM group. The impact of possible phenoconverting drugs (CYP2D6 inhibitors, *n* = 4 or broad enzyme inducers, *n* = 8) was considered negligible at baseline. See Table 1 for a descriptive summary of the cohort.

**Table 1:** Baseline characteristics by CYP2D6 genotype

	Total (*n* = 1554)	NM (*n* = 879/56.6%)	IM + PM (*n* = 675/43.4%)	IM (***n*** = 487/31.3%)	PM (*n* = 188/12.1%)	Missing NM(%)/IM + PM (%)
Age mean (SD) (range)	60.9 (± 11.8) (24*–*94)	60.7 (± 11.8) (24*–*94)	61.2 (± 11.7) (30*–*90)	60.6 (± 11.4) (30*–*90)	62.9 (± 12.4) (31*–*89)	-
Male/female, *n* (%)	1240/314 (79.8/20.2)	694/185 (78.9/21.0)	546/129 (80.9/19.1)	401/86 (82.3/17.7)	145/43 (77.1/22.9)	-
Comorbidities, *n* (%)						
Hypertension	514 (33.1)	289 (32.9)	225 (33.1)	164 (33.7)	61 (32.5)	38 (4.3)/29 (4.3)
Diabetes mellitus	188 (12.1)	101 (11.6)	87 (12.9)	67 (13.8)	20 (10.7)	0/0
Previous myocardial infarction (MI)	215 (13.8)	118 (13.4)	97 (14.3)	74 (15.2)	23 (12.2)	36 (4.1)/28 (4.2)
Stroke	57 (3.7)	32 (3.6)	25 (3.7)	18 (3.7)	7 (3.7)	37 (4.2)/29 (4.3)
Heart failure	26 (1.7)	14 (1.6)	12 (1.8)	8 (1.6)	4 (2.1)	1 (0.1)/0
Renal failure (creatinine > 150 µmol/L)	27 (1.7)	15 (1.7)	12 (1.8)	10 (2.1)	2 (1.1)	0/0
Pharmacotherapy						
Beta-blockers at admission, *n* (%)	343 (22.0)	195 (22.2)	148 (21.9)	113 (23.2)	24 (18.6)	5 (0.6)/8 (1.2)
Metoprolol at discharge, mean dose in mg (SD)	51.5 (± 34.2)	52.7 (± 35.0)	49.6 (± 33.0)	51.1 (± 34.4)	45.8 (± 28.9)	-
STEMI (*n* = 1292)	49.2 (± 31.6)	50.1 (± 32.0)	48.1 (± 31.1)	49.2 (± 31.6)	45.5 (± 29.6)	-
NSTEMI (*n* = 262)	61.7 (± 43.3)	64.7 (± 44.8)	57.5 (± 41.0)	60.1 (± 44.3)	47.8 (± 23.7)	-
If heart failure (*n* = 26)	87.5 (± 55.8)	73.2 (± 47.5)	104.2 (± 62.0)	106 (± 62.3)	100 (± 70.7)	-
If previous MI (*n* = 215)	66.9 (± 47.3)	64.2 (± 43.5)	70.1 (± 51.7)	72 (± 49.8)	61 (± 57.5)	-
If hypertension (*n* = 514)	59.7 (± 40.1)	62.3 (± 41.9)	56.3 (± 37.4)	57 (± 39.2)	54 (± 32.5)	-
CYP2D6 enzyme inhibitors ^a^ at baseline, *n*	4	2	4	4	0	-
Broad enzyme inducers ^b^ at baseline, *n*	8	4	4	4	0	-

Numbers in the table are mean (standard deviation (SD)) or number (%), as specified

^a^Bupropion, fluoxetine, fluvoxamine, paroxetine. ^b^Carbamazepine, phenytoin, phenobarbital

*CYP2D6 *cytochrome p450 enzyme 2D6, *NM *normal metabolizer, *IM *intermediate metabolizer, *PM *poor metabolizer, *STEMI *ST-elevation myocardial infarction, *NSTEMI *non-ST-elevation myocardial infarction

### Primary outcomes

**Fig. 1:**
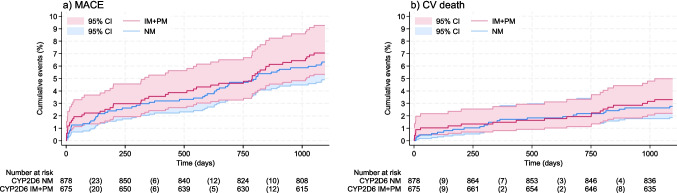
Cumulative incidence plots with risk tables for primary outcomes. **a** Major adverse cardiovascular events (MACE) and **b** cardiosvascular (CV) death. Abbreviations: MACE, major cardiovascular events; CV death, cardiovascular death; CI, confidence interval; CYP2D6, cytochrome p450 enzyme 2D6; NM, normal metabolizer; IM, intermediate metabolizer; PM, poor metabolizer

During the 3-year follow-up, 56 patients (6.4%) in the NM group had an outcome of MACE, and 24 (2.7%) patients suffered from CV death. Corresponding frequency in the IM + PM group was 47 (7.0%) and 22 (3.3%), respectively. There was no association between genotype and MACE [unadjusted HR 1.12 (CI 0.76, 1.65)] or CV death [unadjusted HR 1.20 (CI 0.67, 2.14)] (Fig. 1, Table 2). Similar HRs were seen in adjusted models, as well as in the models using age as the time scale (Table 2).

**Table 2:** Number of events and associations between genotype and clinical outcomes at 3-year follow-up

*Outcome*	Events *n* (%)		Hazard ratio (95% CI) (NM as reference group)				Hazard ratio (95% CI) (NM as reference group) Age as timeline			
	NM *n* = 879	IM + PM *n* = 675	Unadjusted	*p*-value	Adjusted	*p*-value	Unadjusted	*p*-value	Adjusted	*p*-value
*MACE*	56 (6.4)	47 (7.0)	1.12 (0.76,1.65)	0.57	1.12 (0.73,1.68)	0.57	1.10 (0.74,1.63)	0.65	1.10 (0.73,1.66)	0.64
*CV death*	24 (2.7)	22 (3.3)	1.20 (0.67,2.14)	0.53	1.28 (0.69,2.39)	0.42	1.15 (0.64,2.05)	0.64	1.24 (0.66, 2.31)	0.50
*ACM*	44 (5.0)	43 (6.4)	1.27 (0.84,1.95)	0.25	1.45 (0.92,2.28)	0.11	1.26 (0.82,1.93)	0.29	1.04 (0.54,2.01)	0.12
*MI*	10 (1.1)	12 (1.8)	1.57 (0.68,3.63)	0.29	1.59 (0.68,1.67)	0.28	1.69 (0.72,3.93)	0.23	1.69 (0.72,3.95)	0.23
*HF*	12 (1.4)	13 (1.9)	1.42 (0.64,3.12)	0.38	1.37 (0.60,3.10)	0.45	1.32 (0.59,2.96)	0.50	1.27 (0.54,2.98)	0.58
*Stroke*	14 (1.6)	4 (0.6)	0.37 (0.12,1.13)	0.08	0.38 (0.12,1.16)	0.09	0.38 (0.13,1.17)	0.09	0.38 (0.13,1.19)	0.10

CYP2D6 normal metabolizer group vs. intermediate + poor metabolizer group

*CYP2D6 *cytochrome p450 enzyme 2D6, *CI *confidence interval, *NM *normal metabolizer, *PM *poor metabolizer, *MACE *major adverse cardiovascular events, *CV death *cardiovascular death, *ACM *all-cause mortality, *MI *myocardial infarction, *HF *heart failure

### Secondary outcomes

**Fig. 2:**
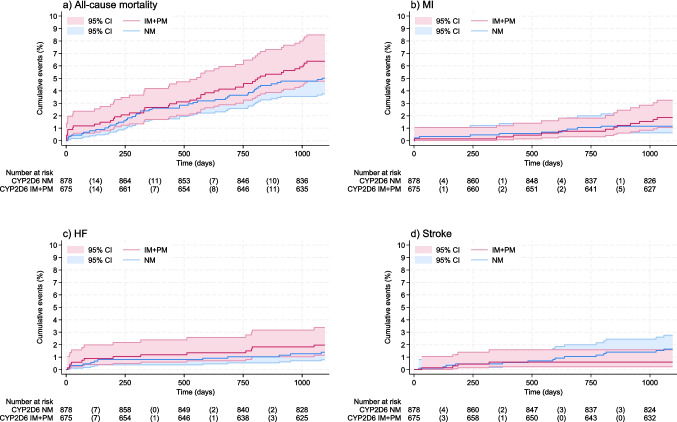
Cumulative incidence plots with risk tables for secondary outcomes. **a** All-cause mortality, **b** myocardial infarction (MI),** c** heart failure (HF), and **d** Stroke. Abbreviations: MACE, major cardiovascular events; CV death, cardiovascular death; CI, confidence interval; CYP2D6, cytochrome p450 enzyme 2D6; NM, normal metabolizer; IM, intermediate metabolizer; PM, poor metabolizer

There was no significant association between *CYP2D6* genotype and all-cause mortality [unadjusted HR 1.27 (CI 0.84, 1.95)], recurrent MI [unadjusted HR 1.57 (CI 0.68, 3.93)], HF [unadjusted HR 1.42 (CI 0.64, 3.12)], or stroke [unadjusted HR 0.38 (CI 0.12, 1.13)] (Fig. 2, Table 2). Adjusted HRs and models using age as the time scale did not change the overall interpretation (Table 2).

### Subgroup analyses

No association was found between genotype and primary or secondary outcomes when comparing the CYPD6 NM and PM subgroup (Table 3), in the subgroups excluding patients treated with metoprolol at baseline ((Supplementary material online, Table S1), when excluding patients with HF at baseline ((Supplementary material online, Table S2), or investigating STEMI ((Supplementary material online, Table S3) and NSTEMI patients separately ((Supplementary material online, Table S4). None of the interaction terms added were significant ((Supplementary material online, Table S5).

**Table 3:** Number of events and associations between genotype and clinical outcomes at 3-year follow-up

*Outcome*	Events *n* (%)		Hazard ratio (95% CI) (NM as reference group)				Hazard ratio (95% CI) (NM as reference group) Age as timeline			
	NM *n* = 879	PM *n* = 188	Unadjusted	*p*-value	Adjusted	*p*-value	Unadjusted	*p*-value	Adjusted	*p*-value
*MACE*	56 (6.4)	16 (8.5)	1.37 (0.79,2.40)	0.27	1.27 (0.72,2.25)	0.40	1.22 (0.70,2.16)	0.48	1.25 (0.70,2.23)	0.46
*CV death*	24 (2.7)	7 (3.7)	1.36 (0.59,3.16)	0.47	1.37 (0.57,3.26)	0.48	1.08 (0.46, 2.56)	0.85	1.24 (0.51,3.05)	0.63
*ACM*	44 (5.0)	12 (6.4)	1.27 (0.67,2.41)	0.46	1.,31 (0.67,2.54)	0.43	1.44 (0.91,2.29)	0.89	1.36 (0.67,2.72)	0.38
*MI*	10 (1.1)	5 (2.7)	2.33 (0.80,6.83)	0.12	2.22 (0.76,6.54)	0.15	2.76 (0.93,8.26)	0.07	2.61 (0.89,7.85)	0.09
*HF*	12 (1.4)	3 (1.6)	1.18 (0.33,4.16)	0.80	0.95 (0.25,3.54)	0.93	1.00 (0.28,3.63)	0.99	1.13 (0.29,4.35)	0.86
*Stroke*	14 (1.6)	2 (1.1)	0.67 (0.15,2.93)	0.59	0.56 (0.13,2.49)	0.45	0.57 (0.12,2.54)	0.46	0.55 (0.12,2.46)	0.43

CYP2D6 normal metabolizer group vs. poor metabolizer group

*CYP2D6 *cytochrome p450 enzyme 2D6, *CI *confidence interval, *NM *normal metabolizer, *PM *poor metabolizer, *MACE *major adverse cardiovascular events, *CV* *death *cardiovascular death, *ACM *all-cause mortality, *MI *myocardial infarction, *HF *heart failure

## Discussion

The present Mendelian randomization study investigated the causal association between CYP2D6 genotype and cardiovascular events among patients treated with metoprolol post-MI. Applying the CYP2D6 metabolizer subgroup as a binary instrument for metoprolol exposure, we were not able to detect an association between genotype and CV death or a composite of MACE among post-MI patients during 3 years follow-up, indicating no beneficial effect of high vs. low dosage of metoprolol.

The just-out results from REDUCE-AMI [15], a prospective open-label RCT, provide the most recent evidence on beta-blocker use post-MI. In this trial, beta-blocker treatment did not ameliorate the risk of death or recurrent MI, contrasting the results from the 3 placebo-controlled studies published in the 1980s [2–4] that demonstrated for the first time that beta-blockers reduce total mortality and sudden cardiac death after MI. Subsequently, beta-blocker treatment has been recommended for secondary prevention post-MI, and the latest European guidelines from 2023 [1] recommend routine beta-blockers to be considered for all acute coronary syndrome patients. However, no target dose is recommended [1] since the RCTs did not assess the effects of different doses of beta-blockers. Applying basic evidence-based medicine principles, the use of beta-blockers that have been studied in randomized trials at the doses used is supported. REDUCE-AMI [15] investigated the beta-blockers metoprolol and bisoprolol, with median doses of 100 mg and 5 mg daily, respectively, and provides important evidence on beta-blocker treatment in patients with preserved left ventricular EF. Nevertheless, the target dose of metoprolol in REDUCE-AMI was half that of the original trial investigating metoprolol [2], which randomized patients to metoprolol 15 mg intravenously followed by oral administration of 100 mg twice daily. In an observational study investigating mortality rate among post-MI patients in relation to the metoprolol dose prescribed at hospital discharge, a 24% 5-year mortality was seen in patients prescribed 200 mg of metoprolol daily, increasing to 43% among patients prescribed 50 mg [34]. On the other hand, in newer observational studies, no benefit of higher vs. lower dose of metoprolol was found [11–14].

Findings from observational studies are challenging to interpret, especially for a drug like metoprolol where age, frailty, blood pressure, and infarction size which have major impact on morbidity and mortality, may affect physicians’ prescription of beta-blockers post-MI. In the study by Herlitz et al., after taking baseline characteristics of age, a history of heart failure, and various complications while in hospital into account, the dose of metoprolol prescribed was not a significant independent predictor of survival. Thus, these results might indicate that the prescription of a high-dose beta-blocker was a good indicator of a low-risk patient more than a mortality benefit of the high-dose treatment.

In the absence of a RCT investigating the importance of achieving a guideline-recommended target dose of metoprolol post-MI, Mendelian randomization can be thought of as analogous to nature’s RCT, considered less susceptible towards confounding and reverse causation than conventional observational studies and that can be used to inform on causation if the underlying assumptions can be justified [25, 26]. We believe that our findings support the results from observational studies indicating no benefit of high vs. low dose of metoprolol after MI. Our findings, however, rely on the assumptions that the genetic instrument used in this study is associated with the exposure, independent of any confounders, and only affects the outcome via the exposure and not through other pathways, that is “no pleiotropy” [25, 27]. We consider the first assumption satisfied, as the association between CYP2D6 genotype and metoprolol previously has been well established [17, 19, 21–23]. We cannot exclude violations of the other underlying assumptions [26, 28, 30], but to our awareness, CYP2D6 is not in linkage disequilibrium with other genetic loci influencing cardiovascular risk through separate pathways [35], and confounding from population stratification would be surprising as our cohort originates from an ethnically homogenous population. Confounding via horizontal pleiotropy could occur if CYP2D6 variants affected the outcome through other pathways than via metoprolol exposure, for instance by involvement in the metabolism of other drugs, and if such drugs influenced cardiovascular risk [27, 30], but this appears an unlikely source of major bias. Overall, we find it reasonable to approve CYP2D6 as a valid instrument for hypothesis testing.

The mean metoprolol dose at discharge in the present study was similar between genotypes. In a previous study, we did not find any difference in the achieved maintenance dose 12 months after MI based on CYP2D6 genotype [17]. In the present study, we did not have prescription data for metoprolol after discharge, but we have previously shown a high compliance of beta-blocker treatment in Norway up to 24 months post-MI, with only minor dose adjustments observed throughout the period [16]. Since there seems to be no clear association between CYP2D6 genotype or metoprolol dose and treatment tolerance [17, 22, 36, 37], a systematic difference between the metabolizer subgroups with respect to treatment discontinuation or compliance is less likely. We therefore assume that most of our patients continued on metoprolol treatment throughout the observation period without significant change of dose. Supported by the increased metoprolol trough plasma concentrations previously demonstrated with the IM and PM genotypes [17], we find it reasonable to assume that we have indeed compared high vs. low exposure of metoprolol.

Overall, our findings do not support that a high vs. low metoprolol exposure is a major determinant of MACE or CV death in post-MI patients. Whether this implies that up-titration to a guideline-recommended target dose of metoprolol has no clinical benefit or that patient characteristics and treatment tolerance should guide individual dosing remains unresolved. As supported in the data from REDUCE-AMI [15], it is also possible that metoprolol regardless of dosage does not improve the long-term outcome among post-MI patients, at least in patients without reduced left ventricular EF. It is noteworthy, however, that 14.1% of patients in REDUCE-AMI randomized to the no beta-blocker group started beta-blocker treatment during the first year of follow-up, while 18.1% in the beta-blocker group stopped treatment. Additional results from upcoming RCTs are expected to resolve this issue [5, 6].

### Strengths and limitations

The main strength was the one-sample design with genetic variants and outcomes obtained from the same individuals. Also, the genotyping panel encompassed a broad range of *CYP2D6* variants, including all variants commonly found in Caucasian populations [38] thereby reducing the risk of erroneous classification of variants as wild type. Although genetic randomization occurs at conception, CYP2D6 genotype is not a measure of lifelong exposure [39], allowing for a defined time zero and reducing the influence of left truncation [32]. Of notion, the onset of exposure could not be accurately defined for the individuals (approximately 20% in both metabolizer groups) already treated with metoprolol upon entering the study; however, subgroup analyses excluding these individuals were consistent with the overall findings.

The study also has some obvious weaknesses. Given the low number of events observed in the cohort, the present findings cannot exclude that a causal effect might exist, but too small to be detected in our study. Moreover, we cannot eliminate the possibility that metoprolol exposure is highly relevant in some individuals, but that the average effect is cancelled by latent subgroups with varying treatment response [26, 31, 40].

Intra-individual variation in CYP2D6 enzyme activity is expected both within the same genotype and within the metabolizer subgroup [29, 39], reducing the strength of CYP2D6 as an IV [31]. Besides, there is no true consensus of CYP2D6 classification into metabolizer subgroups, so both our classification as well as the application of genotype as a binary instrument might have introduced bias. Without information on metoprolol dose, serum concentrations, treatment duration, or compliance, we were only able to investigate the intention-to-treat effect and not the actual effect of metoprolol exposure. Metoprolol exposure could have been modified by several factors, including treatment tolerance, frailty, comorbidities, and drug-drug interactions. Particularly, among NM and IM genotypes concomitant use of drugs inhibiting CYP2D6 could strongly attenuate the effect of genotype, as these genotypes would be converted into PM phenotypes [18, 39]. Nonetheless, even if the exposure could be influenced by other factors, it has been claimed that Mendelian randomization analyses will still be valid [41]. In addition to metoprolol exposure, variation in treatment response could have been induced by polymorphisms in the gene encoding β1-adrenergic receptors [37], but these variants were not included in the present study.

Also, the Cox model comes with some limitations. It assumes the HR to be constant over time, so the estimated HRs are averaged over, and therefore dependent on the duration of follow-up. Moreover, assessing the PH assumption is difficult when number of events is low and is expected to hold for either the unadjusted or the adjusted model [33, 40]. Due to the non-collapsibility of the HR, only population-level HRs could be assessed. The model assumes uninformative censoring, so as death as a competing event was censored the tested hypotheses are cause-specific [40].

## Conclusion

In this one-sample, individual-level Mendelian randomization study using *CYP2D6* genotype as a proxy for metoprolol exposure, we were not able to detect any influence of CYP2D6 metabolizer subgroup on the occurrence of cardiovascular events and death in patients treated with metoprolol following MI during 3 years of follow-up.

## Supplementary Information

Below is the link to the electronic supplementary material.

ESM 1 (PDF 708 KB)

## Data Availability

The data underlying this article will be shared on reasonable request to the corresponding author.
