# Supplementary material for: Effect of metoprolol exposure following myocardial infarction on future cardiovascular events: a Mendelian randomization study
Source: Eur J Clin Pharmacol. 2025 Feb 3;81(4):551–60. doi: 10.1007/s00228-025-03806-w (PMC11923007; doi:10.1007/s00228-025-03806-w)
Supplement: Supplementary file 1 — (PDF 708 KB) [file 228_2025_3806_MOESM1_ESM.pdf]

Supplemental material

Title: Effect of metoprolol exposure following myocardial infarction on future cardiovascular events: a Mendelian randomization study

Journal: European Journal of Clinical Pharmacology

Lina Dorthea Bruun[1], Geir Øystein Andersen[2], Marianne Kristiansen Kringen[3,4], Peder Langeland Myhre[5,6], Sigrun Halvorsen[2,7], Charlotte Holst Hansen[2], Espen Molden[3,8], Erik Øie[7,9]

[1]Department of Pharmacology, Oslo University Hospital, Ullevål, Oslo, Norway

[2]Department of Cardiology, Oslo University Hospital, Ullevål, Oslo, Norway

[3]Center for Psychopharmacology, Diakonhjemmet Hospital, Oslo, Norway.

[4]Department of Health Sciences, Oslo Metropolitan University, Oslo, Norway.

[5]Division of Medicine, Akershus University Hospital, Lørenskog, Norway

[6]K.G. Jebsen Center of Cardiac Biomarkers, Institute for Clinical Medicine, University of Oslo, Oslo, Norway

[7]Institute for Clinical Medicine, University of Oslo, Oslo, Norway

[8]Department of Pharmacy, University of Oslo, Oslo, Norway.

[9]Department of Internal Medicine, Diakonhjemmet Hospital, Oslo, Norway

Corresponding Author:

Lina Dorthea Bruun, MD

Department of Pharmacology, Oslo University Hospital, Ullevål,

PO BOX 4956 Nydalen, N-0424 Oslo, Norway

linabruun@yahoo.no

List of supplemental tables and figures

Supplemental Table S1 - Excluding patients on beta-blocker treatment at baseline. Number of events outcomes.

Supplemental Table S2 - Excluding patients with heart failure at baseline. Number of events and outcomes.

Supplemental Table S3 - ST-elevation myocardial infarction (STEMI) patients only. Number of events outcomes.

Supplemental Table S4 - Non ST-elevation myocardial infarction (NSTEMI) patients only. Number of events and outcomes.

Supplemental Table S5 - Gene-covariate interaction for primary outcomes.

Supplemental Figure S1 – Study flow chart.

Table S1. Number of events and associations between CYP2D6 metabolizer group and clinical outcomes at 3 year follow-up. Subgroup excluding patients on beta-blocker treatment at baseline.

Outcome	Events n (%)		Hazard ratio (95% CI) (NM as reference group)			
	NM n=684	IM+PM n=527	unadjusted	p-value	adjusted	p-value
MACE	35 (5.1)	34 (6.5)	1.30 (0.81,2.10)	0.27	1.31 (0.81,2.13)	0.27
CV death	14 (2.0)	13 (2.5)	1.21 (0.57,2.57)	0.62	1.34 (0.61,2.92)	0.46
ACM	26 (3.8)	25 (4.7)	1.25 (0.72,2.17)	0.42	1.41 (0.79,2.50)	0.24
MI	8 (1.2)	12 (2.3)	1.95 (0.80,4.78)	0.14	2.01 (0.82,4.92)	0.13
HF	6 (0.9)	11 (2.1)	2.40 (0.89,6.5)	0.08	2.46 (0.86,6.98)	0.09
Stroke	9 (1.3)	2 (0.4)	0.28 (0.06,1.33)	0.11	0.29 (0.06,1.35)	0.11

Abbreviations: CYP2D6, cytochrome p450 enzyme 2D6; CI, confidence interval; NM, normal metabolizer; PM, poor metabolizer; MACE, major adverse cardiovascular events; CV death, cardiovascular death; ACM, all-cause mortality; MI, myocardial infarction; HF, heart failure.

Table S2. Number of events and associations between CYP2D6 metabolizer group and clinical outcomes at 3 year follow-up. Excluding patients with heart failure at baseline.

Outcome	Events n (%)		Hazard ratio (95% CI) (NM as reference group)			
	NM n=725	IM+PM n=568	unadjusted	p-value	adjusted	p-value
MACE	28 (3.9)	28 (4.9)	1.29 (0.76, 2.17)	0.34	1.32 (0.78, 2.27)	0.30
CV death	8 (1.1)	9 (1.6)	1.44 (0.56, 3.74)	0.45	1.74 (0.64, 4.71)	0.27
ACM	21 (2.9)	23 (4.4)	1.41 (0.78, 2.54)	0.26	1.84 (0.96, 3.52)	0.06
MI	8 (1.1)	11 (1.9)	1.77 (0.71, 4.39)	0.22	1.80 (0.72, 4.45)	0.21
HF	2 (0.3)	6 (1.1)	3.87 (0.78,19.16)	0.10	3.29 (0.63, 17.17)	0.16
Stroke	11 (1.5)	4 (0.7)	0.47 (0.15, 1.46)	0.19	0.52 (0.16, 1.64)	0.26

Abbreviations: CYP2D6, cytochrome p450 enzyme 2D6; CI, confidence interval; NM, normal metabolizer; PM, poor metabolizer; MACE, major adverse cardiovascular events; CV death, cardiovascular death; ACM, all-cause mortality; MI, myocardial infarction; HF, heart failure.

Table S3. Number of events and association between CYP2D6 metabolizer group and clinical outcomes at 3 year follow-up. ST-elevation myocardial infarction (STEMI) patients only.

Outcome	**Events n (%)**		**Hazard ratio (95% CI) (NM as reference group)**			
	**NM n=725**	**IM+PM n=567**	**unadjusted**	**p-value**	**adjusted**	**p-value**
MACE	45 (6.2)	36 (6.3)	1.04 (0.67,1.63)	0.84	1.01 (0.69,1.68)	0.75
CV death	20 (2.8)	16 (2.8)	1.02 (0.53,1.97)	0.95	1.07 (0.55,2.11)	0.82
ACM	35 (4.8)	32 (5.6)	1.20 (0.74,1.95)	0.45	1.28 (0.78,2.10)	0.32
MI	9 (1.2)	10 (1.8)	1.42 (0.58,3.49)	0.45	1.43 (0.58,3.52)	0.43
HF	9 (1.2)	10 (1.8)	1.42 (0.58,3.51)	0.44	1.60 (0.62,4.10)	0.33
Stroke	11 (1.5)	4 (0.7)	0.46 (0.15,1.46)	0.18	0.48 (0.15,1.53)	0.22

Abbreviations: CYP2D6, cytochrome p450 enzyme 2D6; CI, confidence interval; NM, normal metabolizer; PM, poor metabolizer; MACE, major adverse cardiovascular events; CV death, cardiovascular death; ACM, all-cause mortality; MI, myocardial infarction; HF, heart failure.

Table S4. Number of events and associations between CYP2D6 metabolizer group and clinical outcomes at 3 year follow-up. Non ST-elevation myocardial infarction (NSTEMI) patients only.

Outcome	**Events n (%)**		**Hazard ratio (95% CI) (NM as reference group)**			
	**NM n=154**	**IM+PM n=108**	**unadjusted**	**p-value**	**adjusted**	**p-value**
MACE	11 (7.1)	11 (10.2)	1.48 (0.64, 3.41)	0.36	1.49 (0.55, 4.01)	0.43
CV death	4 (2.6)	6 (5.6)	2.20 (0.62, 7.78)	0.22	3.83 (0.61, 24.23)	0.15
ACM	10 (6.5)	11 (10.2)	1.62 (0.69, 3.80)	0.27	3.32 (0.90,12.22)	0.07
MI	1 (0.6)	2 (1.9)	2.96 (0.27,32.64)	0.38	2.96 (0.27,32.80)	0.38
HF	3 (1.9)	3 (2.8)	1.48 (0.29,7.33)	0.63	0.79 (0.10, 6.02)	0.16
Stroke	3 (1.9)	0 (0)	-	-	-	-

Abbreviations: CYP2D6, cytochrome p450 enzyme 2D6; CI, confidence interval; NM, normal metabolizer; PM, poor metabolizer; MACE, major adverse cardiovascular events; CV death, cardiovascular death; ACM, all-cause mortality; MI, myocardial infarction; HF, heart failure.

Table S5. Gene-covariate interaction for primary outcomes.

	p-value of gene-covariate interaction	
**Covariate**	**MACE**	**CV death**
Sex	0.31	0.77
Age	0.58	1.00
Type of MI (STEMI/NSTEMI)	0.48	0.98
HF at baseline*	0.54	0.74
Diabetes mellitus	0.81	0.98

* a composite of established HF and/or serum N-terminal pro-B-type natriuretic peptide (NT-proBNP) >500 ng/mL and/or left ventricular ejection fraction <40% at baseline

Abbreviations: MACE, major cardiovascular events; CV death, cardiovascular death; MI, myocardial infarction; STEMI, ST-elevation myocardial infarction; NSTEMI, non ST-elevation myocardial infarction; HF, heart failure.

Fig S1. Study flow chart

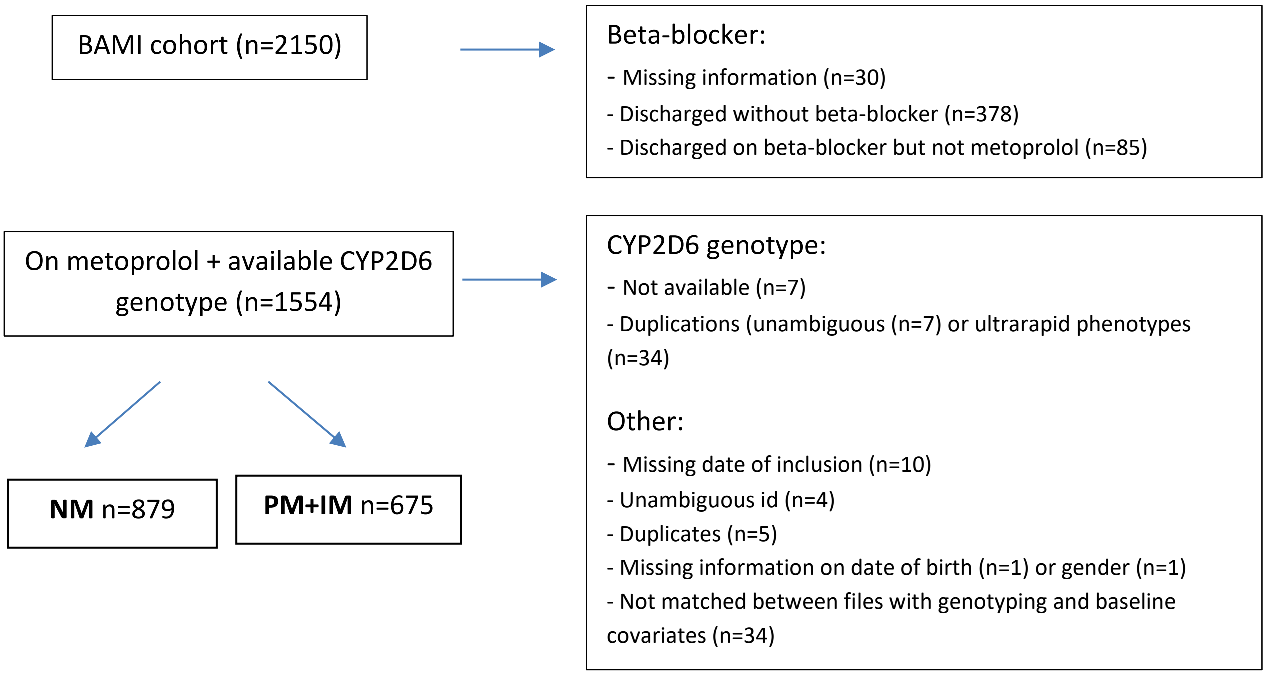

Abbreviations: BAMI, Biobanking in Acute Myocardial infarction; CYP2D6, cytochrome p450 enzyme 2D6; NM, normal metabolizer; IM, intermediate metabolizer; PM, poor metabolizer.
